# The Development, Acceptability and Suitability of an Information and Well‐Being Booklet for Family Members of Intensive Care Unit Patients

**DOI:** 10.1111/nicc.70589

**Published:** 2026-07-20

**Authors:** Lila Simpson, Malaika Subhani, Shamini Satkunam, Sylvia Puchalska, Madiha Shaikh

**Affiliations:** ^1^ Department of Psychology Royal Holloway, University of London Egham UK; ^2^ Department of Medicine Trinity College, University of Cambridge Cambridge UK; ^3^ Clinical Health Psychology North East London Foundation NHS Trust London UK; ^4^ Critical Care Queen's Hospital, Barking, Havering and Redbridge University Hospitals Trust London UK; ^5^ Research Department of Clinical Educational and Health Psychology University College London London UK

**Keywords:** carers, critical care, families, family, ICU, support, well‐being

## Abstract

**Background:**

Intensive Care Unit (ICU) admission is a highly stressful and often unexpected experience for family members, frequently resulting in significant psychological distress and ongoing caregiving burden. Despite widespread awareness and recognition of these needs, structured psychosocial interventions for family members remain limited. In response, a family well‐being intervention was developed within a UK ICU psychology service, incorporating a clinician‐delivered psychoeducational and support programme alongside an information and well‐being booklet. This study evaluated the booklet prior to implementation.

**Aims:**

To explore family members' perceptions of the newly developed ICU information and well‐being booklet, focusing on its usefulness, accessibility and acceptability and to inform refinement prior to clinical implementation.

**Study Design:**

A qualitative design was adopted. Semi‐structured interviews were conducted with five family members of patients previously admitted to ICU in a London hospital. Participants were purposively sampled and reviewed the booklet during interviews conducted via Microsoft Teams. Data were analysed using reflexive thematic analysis.

**Findings:**

Four overarching themes were identified: (1) Information as Emotional Containment and Orientation; (2) Communication as Containment and Validation; (3) Preparation for Transitions and Recovery Beyond ICU; and (4) The Overlooked Burden: Acknowledging Carers' Well‐being. Participants described the booklet as accessible, clear and emotionally containing. It was perceived to reduce uncertainty, validate emotional responses, support communication with staff and provide anticipatory guidance regarding recovery and transitions. Family members particularly valued explicit recognition of caregiver distress and permission to prioritise self‐care.

**Conclusions:**

The ICU psychoeducation and well‐being booklet was perceived as a useful and acceptable resource that addresses both informational and psychological needs of family members.

**Relevance to Clinical Practice:**

Beyond information provision, findings suggest the booklet may function as a psychologically containing and orienting tool during periods of acute uncertainty.

## Impact Statement

- What is known about this topic
  ○ Psychological Burden: Intensive Care Unit (ICU) admission is an acute, distressing experience that routinely subjects family members to severe anxiety, depression and long‐term caregiver burnout.
  ○ The Implementation Gap: While the literature extensively documents the profound psychological and informational needs of ICU families, structured, evidence‐based psychosocial interventions remain scarce, variable in quality and poorly integrated into standard care.
  ○ Passive Information Limitations: Traditional ICU leaflets focus primarily on medical jargon or basic ward orientation, rarely addressing the emotional containment, coping mechanisms or transitional care needs of the family as an interdependent unit.
- What this paper adds
  ○ Co‐produced, framework‐driven resource: This study evaluates a novel, cognitive‐behavioural framework‐driven information and well‐being booklet—developed within a UK NHS psychology service—designed as a core component of a brief, 8‐session clinician‐led intervention.
  ○ Information as emotional containment: Qualitative evaluation via reflexive thematic analysis reveals that the booklet operates as a tool for psychological containment. Rather than just offering facts, it actively reduces acute uncertainty, normalises emotional trauma and validates the overlooked burden of the caregiver.
  ○ Anticipatory guidance for recovery: The findings demonstrate the high acceptability and utility of explicit, step‐by‐step guidance on communicating with ICU staff and preparing for the complex physical and psychological transitions beyond the ICU walls.
  ○ Pre‐implementation refinement: By capturing the lived experience of families who have navigated the ICU environment, this paper provides a highly practical model for refining digital and physical psychoeducational materials prior to full clinical trials.

## 1 Introduction

Intensive care units (ICUs) are high‐stress, complex and potentially traumatic environments for both patients and their families. ICU admission is frequently unplanned, confronting family members with unexpected responsibility, complex decision‐making and uncertainty regarding their relative's condition and prognosis [1]. During this period, family members often experience intense anxiety, fear of deterioration or death, disruption to family roles and significant emotional distress [2, 3].

The psychological impact of an ICU admission frequently extends beyond hospitalisation. Following discharge, family members often assume informal caregiving responsibilities, likely further exacerbating the emotional and psychological burden. In recognition of this, the term Post Intensive Care Syndrome‐Family (PICS‐F) has been introduced to describe the psychological, emotional and social difficulties experienced by family members of critically ill patients [4, 5]. It specifically highlights the heightened risk of depression, anxiety, post‐traumatic stress disorder (PTSD) and complicated grief, which may arise during ICU admission and persist long after discharge [6, 7, 8].

There is increasing recognition of the need for targeted, person‐centred support for family members during and after ICU admission. Research consistently identifies key areas of need. These include clear, timely and compassionate communication, alongside accessible and understandable information about the ICU environment, treatment processes and prognosis [1, 9, 10]. In addition, family members report a need for emotional reassurance, validation and access to psychosocial support during and post ICU admission [10]. Information provision in ICU contexts may function not only to increase understanding, but also to reduce uncertainty, restore a sense of agency, support emotional containment and facilitate coping during periods of acute stress.

Despite a robust evidence base documenting the needs and experiences of family members, interventions designed specifically to support family members of patients in ICU remain limited, variable in quality and inconsistent across services [11, 12, 13]. These findings highlight a clear gap between the needs identified in the literature and the support currently available.

To address this, we have developed a family well‐being intervention designed specifically to support family members of patients admitted to ICU. The intervention is informed by a cognitive behavioural framework and is designed to be delivered by a member of the ICU psychology team over eight individual sessions for brevity. It includes psychoeducation, individualised well‐being support and signposting to relevant services. A central component of the intervention is an information and well‐being booklet, designed to provide accessible information about the ICU environment, normalise common emotional responses, offer practical coping strategies and signpost support during and after admission. It is intended to be used alongside the clinician‐led sessions, providing a structured resource that family members can use and revisit at their own pace. The present study focuses specifically on evaluating the booklet to evaluate its accessibility, relevance and acceptability prior to embedding it within the wider intervention.

## 2 Aims

The primary aim of this study is to explore family members' perceptions of the newly designed information and well‐being booklet (see Supporting Information), focusing on its usefulness, accessibility and acceptability. Findings will inform refinement of the booklet to ensure it is meaningful, accessible and suitable for implementation alongside the intervention within our service. Specifically, the study aims to:

1. Explore family members' views on the usefulness of the booklet in supporting their understanding of the ICU environment and journey and emotional well‐being during and/or after ICU admission.
2. Explore the accessibility and clarity of the information provided within the booklet.
3. Use family member feedback to refine and adapt the booklet prior to its implementation within the wider intervention.

## 3 Methodology

### 3.1 Service Setting

This study was conducted on six ICU wards, all based in a London hospital in 2025. These consisted of two general ICUs, one long‐stay ICU, one elective surgery ICU, one High dependency unit and one specialist Neuro‐ICU.

### 3.2 Development of the Well‐being Booklet

#### 3.2.1 Phase 1: Identifying the Specific Needs of Family Members

The first phase aimed to identify the key needs of family members of ICU patients. This was informed by a previous project conducted within our service [14], involving semi‐structured interviews with five family members of patients discharged from ICU. Thematic analysis identified key needs in three areas:

1. Communication: information communicated in a human‐centred, compassionate and accessible manner, with minimal use of medical jargon.
2. Information: need for understandable explanations regarding ICU equipment, diagnosis, treatment and prognosis.
3. Psychosocial support: emotional reassurance and clear signposting to support services.

These findings were supplemented by an in‐depth review of the literature, which similarly highlighted the need for accessible information, clear communication, emotional reassurance and inclusion of family as part of the ICU team.

#### 3.2.2 Phase 2: Booklet Development

An initial draft of the booklet was developed using the findings from phase 1. The draft included several chapters designed to provide: accessible information about the ICU environment, what to expect when transitioning from ICU to the wards and then to discharge, coping strategies and signposting to support services.

A consultation meeting was held with five members of the ICU Psychology team to obtain feedback on the initial draft. Feedback was collated and reviewed, and revisions were made based on this. The second draft was reviewed again by the lead psychologist, with no further amendments required.

The booklet chapters and contents are summarised in Table 1 and the booklet is provided in Supporting Information.

**TABLE 1:** Contents of the booklet.

Section	Contents
Introduction	Outlines aims and purpose of the booklet.
The intensive care unit (ICU)	Explains what ICU is and common reasons for being there.
Equipment in ICU	Outlines most common pieces of equipment in ICU—explains their role and function and provides pictures.
Key people in ICU	Outlines the role of key members of staff in ICU.
Communication	Discusses the best ways to communicate with ICU staff and provides a hierarchy of who best to contact first. Provides information on use of interpreters.
Delirium	Provides psychoeducation on delirium: what it is, causes, likely duration and how family members can help.
Leaving ICU	Discusses what to expect when moved to another ward.
Going home and recovery	Details what to expect for recovery and common difficulties. Advice on how family members can support their loved one. Information on ICU follow up process.
Well‐being tips and strategies	Provides useful strategies to cope with common difficulties (sleep problems, anxiety, low mood) and support well‐being.
End of life	Discusses how the team know someone is at the end of their life, how family members can support at the end, how family members can get support, information on grief and specific signposting to bereavement services.
Support and help directory	Details sources of further help and support services.

### 3.3 The Broader Family Well‐being Intervention

The booklet forms part of a wider low‐intensity well‐being intervention delivered over eight individual sessions. The intervention comprises three components:

1. Psychoeducation: family members read through the information booklet together with their clinician, pausing after each section to reflect on the relevance to their own experience and asking any questions.
2. Well‐being support: tailored, idiosyncratic support considering the impact of the ICU admission and/or their caring role on anxiety, mood, self‐care, trauma symptoms and quality of life. Clinicians may draw on a number of low‐intensity interventions, including problem‐solving, sleep hygiene, time management or behavioural activation, depending on the family member's goals and stage in their ICU journey.
3. Well‐being planning and signposting: consolidation of learning, relapse prevention and guidance on further support services.

### 3.4 Design

A qualitative design using reflexive thematic analysis [15] was adopted within a critical realist framework. This approach was considered appropriate given the study aimed to explore family members' subjective experiences and meanings while recognising that these accounts are shaped by broader social and healthcare contexts.

### 3.5 Participants

Participants were family members of patients previously admitted to an ICU based in London. Family members of patients still in ICU were not considered to avoid adding to the high levels of stress and emotional burden experienced during an active ICU admission.

Purposive sampling was used. Participants were identified through consultation with the ICU multidisciplinary team (MDT), with the aim of recruiting a diverse sample of family members across a range of ages, relationships to the patient and ethnic backgrounds. Inclusion criteria were: being aged 18 years or older, identified as a primary caregiver or close relative of a patient discharged from ICU and able to read and understand English. Exclusion criteria included: family members of patients still admitted to ICU; individuals unable to engage with the booklet due to language or cognitive barriers. Sample size was guided by the study's focused evaluative aims and the depth of experiential data generated, rather than assumptions of thematic saturation.

### 3.6 Data Collection

Data were collected using semi‐structured interviews conducted online via Microsoft Teams. Basic demographic and contextual information was collected prior to the interview.

During the interview, participants were provided with an electronic version of the booklet via screen sharing on Microsoft Teams. The interviewer and participant reviewed the booklet together, discussing each section in detail. An interview topic guide was used to prompt open‐ended discussion about participants' perceptions of the booklet. Questions focused on participants' overall impressions, clarity and accessibility of the content and acceptability of the booklet as a support resource. Interviews lasted approximately 45–60 min and were audio‐recorded and transcribed verbatim for analysis.

### 3.7 Data Analysis

Data were analysed using thematic analysis [16]. The analysis followed the six phases described by Braun and Clarke [16]: (1) familiarisation with the data, (2) generation of initial codes, (3) searching for themes, (4) reviewing themes, (5) defining and naming themes and (6) producing the report.

Consistent with a reflexive thematic analysis approach, the lead researcher acknowledged their active role in interpreting the data and viewed subjectivity as an analytic resource [17, 18]. The lead researcher was a White British, middle‐class, non‐religious, cis‐gendered, female Trainee Clinical Psychologist, who was completing a placement within the ICU psychology service where the study was conducted. She had previous experience delivering psychological support to family members in ICU and was involved in the development of the booklet. It was therefore critical to remain conscious of how both her personal and professional background and experiences may shape assumptions about what appropriate family support in ICU ‘should’ look like and therefore influence interpretation of the data. Reflexivity was therefore maintained throughout the study through collaborative discussions with the research assistant and lead psychologist. Differences in our professional roles, personal backgrounds and demographic characteristics provided opportunities to challenge assumptions, sense‐check interpretations and consider alternative perspectives throughout the analytic process [17, 18].

Data were analysed at the semantic level and analyses adopted a primarily inductive orientation, meaning themes were generated from participants' accounts rather than informed by pre‐existing theoretical frameworks or predefined components of the booklet [19]. This enabled family members to emphasise aspects of the booklet they perceived as most meaningful, supporting the generation of family‐led insights relevant to the refinement and future implementation of the booklet.

Initial coding was undertaken by the primary researcher (LS), with a second researcher (MSu) independently reviewing selected transcripts to facilitate reflexive dialogue, challenge assumptions and deepen interpretative engagement with the data. Emerging themes were then discussed collaboratively, including consultation with the lead Clinical Psychologist (MS) of the service, to sense‐check interpretations and explore alternative assumptions. Discussions focused on exploring alternative interpretations rather than establishing coding reliability or consensus [19].

### 3.8 Ethical Considerations

The project was approved through the hospital's clinical service evaluation governance procedures. The study aimed to evaluate and refine an internally developed clinical resource within routine service development activities and therefore did not require NHS Research Ethics Committee review under HRA guidance. Nonetheless, steps were taken to ensure the study adhered to ethical standards and protected participant well‐being. Participants received an information sheet outlining the study aims, procedures and dissemination plans. They were informed that participation was voluntary, that they could withdraw at any time without explanation or consequence and that data would be treated confidentially. Verbal informed consent was obtained prior to participation.

## 4 Findings

### 4.1 Participant Characteristics

A total of 5 family members took part in this study, comprising one male and four females. Participant characteristics are summarised in Table 2.

**TABLE 2:** Participant characteristics.

	Gender	Age	Ethnicity
Ppt 1	Female	31	White British
Ppt 2	Female	52	White British
Ppt 3	Female	42	White non‐British
Ppt 4	Male	85	White British
Ppt 5	Female	39	White British

### 4.2 Themes

**FIGURE 1:**
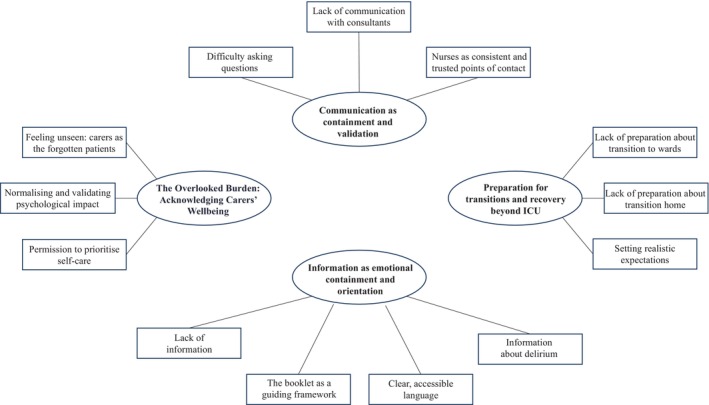
Map of themes and sub themes.

Analysis identified four main overarching themes, each with related sub themes, capturing family members' perceptions of the information and well‐being booklet, as shown in Figure 1. Supporting quotations for subthemes are provided in Supporting Information.

#### 4.2.1 Theme 1: Information as Emotional Containment and Orientation

Family members described ICU admission as highly disorientating, characterised by uncertainty, confusion and emotional distress. A perceived lack of accessible information intensified these experiences and contributed to feelings of loss of control. The booklet was valued as a structured and psychologically containing resource that enhanced understanding and provided orientation within the ICU environment.

##### 4.2.1.1 Lack of Information

Participants highlighted the distress associated with not understanding ICU processes or equipment:

Not knowing what to expect… it's overwhelming…why is that beeping? Why is that making noise? (P1).

You feel so out of control (P3).

##### 4.2.1.2 The Booklet as a Guiding Framework

The booklet was experienced as a practical framework that could guide understanding and facilitate further discussion with staff:

It gives you a guide of what's gonna be happening (5).

This would be enough information and then you could go and ask more if you needed to after this (2).

##### 4.2.1.3 Clear, Accessible Language

Participants also valued the use of clear, accessible language and absence of excessive medical jargon within the booklet:

It's using simple language to describe a difficult task (4).

It doesn't feel like it's professional (5).

##### 4.2.1.4 Information About Delirium

Information regarding ICU delirium was perceived as particularly important and reassuring, especially given participants' prior lack of awareness:

I'm glad you've put this in because …I wasn't told about any of this. (2).

It's important to mention right at the beginning that it's common for patients in ICU to begin to experience delirium. So you're instantly reassured (3).

#### 4.2.2 Theme 2: Communication as Containment and Validation

Participants described communication within ICU as difficult and, at times, inaccessible. Families often felt uncertain about whether they were entitled to ask questions or seek clarification, particularly within the pressured ICU environment. The booklet was perceived as validating these difficulties and supporting communication with staff.

##### 4.2.2.1 Difficulty Asking Questions

Participants described hesitancy in approaching staff due to fears of interrupting clinical work:

You might have a question but fail to ask it because you're terrified you might stop them (consultants and nurses) from doing their job (4).

##### 4.2.2.2 Lack of Communication With Consultants

Communication with consultants was frequently experienced as brief and rushed:

I wanted to talk to the consultant, but sometimes they would be so rushed and it would just be like 2 min of an update and I would be disappointed (3).

##### 4.2.2.3 Nurses as Consistent and Trusted Points of Contact

In contrast, nurses were consistently described as approachable and trusted sources of support and explanation:

The intensive care nurses are very knowledgeable…I found that without them, I don't think I would have got by in there. They're the ones that explain (2).

#### 4.2.3 Theme 3: Preparation for Transitions and Recovery Beyond ICU

Transitions from ICU to the ward and subsequently home were commonly experienced as abrupt and anxiety provoking. Participants described feeling underprepared for the reduction in support following ICU discharge, with some experiencing a sense of abandonment or discontinuity of care. The booklet was perceived as helping families anticipate these transitions and develop more realistic expectations regarding recovery.

##### 4.2.3.1 Lack of Preparation About Transition to Wards

Participants described the transition to the ward as particularly difficult and felt the information highlighting this in the booklet would have helped them prepare and reduced stress:

We didn't know any of this. We just felt a massive change…Once you're on a ward, you kind of get forgotten about (2).

A big adjustment… hearing this initially would be helpful and less stressful (3).

##### 4.2.3.2 Lack of Preparation About Transition Home

Similarly, families felt insufficiently prepared for discharge home:

I'm glad you put that in there because I had to ring up and ask for help because I didn't really know what to do (2).

The after bit is really lovely because that's something we knew nothing about (4).

##### 4.2.3.3 Setting Realistic Expectations

Participants also valued information normalising the non‐linear nature of recovery:

It needs to be explained that it's not going to be as easy as you think… thought he was going to come home and thought he was going to carry on as normal and it didn't go like that (2).

#### 4.2.4 Theme 4: The Overlooked Burden: Acknowledging Carers' Well‐being

Family members described significant emotional and physical strain associated with supporting a relative in ICU, alongside feelings of invisibility within systems primarily focused on the patient. The booklet was experienced as legitimising caregiver distress, validating emotional responses and granting permission for self‐care.

##### 4.2.4.1 Feeling Unseen: Carers as the Forgotten Patients

Participants described feeling overlooked despite experiencing substantial psychological burden. They felt that the booklet could help acknowledge and validate these experiences:

I would like this booklet to kind of have a feel of, carers are important and we see you…because you're not just the carer… you end up being a patient yourself but it gets missed…You end up struggling on an emotional level and a physical level…but just because you're not on the bed you can't be just dismissed (3).

##### 4.2.4.2 Normalising and Validating the Psychological Impact

Emotional distress was commonly described and participants valued the booklet's normalisation of these reactions:

It was the mental bit that got me…you can really go downhill mentally when you are put into this sort of situation (4).

It's okay not to be okay (2).

##### 4.2.4.3 Permission to Prioritise Self‐Care

Feelings of guilt regarding time away from the bedside were also prominent. Participants described the importance of explicitly encouraging carers to prioritise their own well‐being:

You feel like you need to be there 24/7…you don't have to be (2).

Be kind to yourself. Don't be too hard on yourself… You're only human, you know. You can only do what's possible (4).

### 4.3 Wider Recommendations

Participants also raised broader suggestions beyond the scope of the booklet. Two participants recommended leaving brief written summaries at the bedside following ward rounds to reduce pressure on families to attend at specific times, minimise anxiety about missing information and improve involvement in care and treatment decisions.

### 4.4 Refinements Made to Booklet

Themes and subthemes were discussed with the research assistant and lead psychologist, informing final refinements to the booklet. Refinements included:

- Highlighting the role of the booklet as a guiding framework to support understanding and encourage families to seek further information as needed
- Explicitly encouraging family members to ask questions, acknowledging challenges in communicating with consultants and more clearly emphasising nurses as key points of contact.
- Within the transition and recovery section, content was expanded to emphasise the non‐linearity of recovery and the importance of celebrating small wins.
- Common emotions experienced by family members, including guilt, exhaustion, anger and resentment, were more explicitly highlighted and normalised.
- A paragraph was added to explicitly highlight the importance of, and grant permission for, family members to prioritise their own well‐being and self‐care.
- Signposting to support services was made clearer, with more explicit guidance on how and when to access available support.

## 5 Discussion

This study explored family members' perceptions of a newly developed information and well‐being booklet designed to support relatives of patients admitted to ICU. The booklet forms one component of a wider family well‐being intervention developed within the service and was evaluated to assess perceived usefulness, accessibility and acceptability prior to implementation. Reflexive thematic analysis identified four core themes: information as emotional containment and orientation; communication as containment and validation; preparation for transitions and recovery beyond ICU; and the overlooked burden of acknowledging carers' well‐being. Collectively, findings suggest that the booklet operates beyond a traditional psychoeducational resource, functioning as a psychologically containing intervention by reducing uncertainty, legitimising emotional responses, supporting communication and enhancing families' sense of agency during a period of profound disruption.

A consistent finding was that uncertainty and lack of understanding contributed significantly to family distress during ICU admission. This aligns with previous research demonstrating that unmet information needs are common among ICU relatives and are associated with increased anxiety and emotional burden [20, 21]. Consistent with guidance on family‐centred critical care, timely and comprehensible information is essential in supporting relatives' understanding and involvement [22]. Participants particularly valued the booklet's accessible language and practical explanations, suggesting that written resources may complement verbal communication by providing families with a stable reference point that can be revisited when cognitive and emotional capacity is reduced by stress.

Communication difficulties were also prominent, with participants describing uncertainty about how and when to approach staff, particularly within the highly specialised and hierarchical ICU environment. Similar barriers have been described across healthcare settings, where perceived power differences can inhibit families from voicing concerns or seeking clarification [23, 24, 25]. In this study, the booklet appeared to act as a communication scaffold by explicitly legitimising questions and clarifying routes for support. Participants' emphasis on nurses as trusted and consistent sources of information reinforces the central role of nursing teams in facilitating family engagement and providing relational support within ICU care [26].

Transitions from ICU to ward‐based care and discharge home were experienced as particularly challenging, with participants describing these stages as abrupt changes associated with reduced support and uncertainty about recovery. This reflects wider literature identifying transitions in critical care as periods of heightened vulnerability due to discontinuity of information and support [27, 28, 29]. The booklet was valued for preparing families for the non‐linear nature of recovery and helping to establish realistic expectations. By maintaining informational continuity across transitions, the resource may help reduce the psychological impact of moving between care environments.

A further important finding was the need to recognise family members as recipients of care in their own right. Participants described substantial emotional strain, including guilt, exhaustion, anxiety and feeling overlooked while attention remained focused on the patient. This reflects evidence demonstrating high levels of psychological morbidity among ICU relatives, including anxiety, depression and trauma‐related symptoms [6, 7]. The booklet's explicit acknowledgement of these experiences was perceived as validating and normalising, suggesting that family interventions should extend beyond information provision to address emotional well‐being and self‐care.

Participants also described neglecting their own needs during ICU admission, including reduced sleep, limited self‐care and sustained emotional demands. This is clinically important given evidence that stress and fatigue may impair information processing, decision‐making and longer‐term psychological well‐being [30, 31, 32]. The findings therefore support the inclusion of family well‐being interventions within critical care pathways, particularly during periods when relatives may be least able to identify or advocate for their own support needs.

### 5.1 Limitations

The sample was small and predominantly comprised White British, English‐speaking participants, limiting transferability to more diverse populations. Existing evidence indicates that factors including ethnicity, socioeconomic status, gender, education and cultural background influence ICU experiences and support needs [1, 3, 33]. Future research should prioritise more diverse recruitment and consider culturally and linguistically adapted versions of the booklet.

Data collection and analysis were undertaken by clinicians involved in booklet development, introducing potential for interpretative bias. Although efforts were made to encourage critical feedback and support reflexive discussion, the dual role of clinician and researcher should be considered when interpreting findings.

Although resources such as ICUsteps provide valuable information regarding ICU recovery, the present intervention differs through its explicit focus on the psychological needs of family members and its integration within a clinician‐led well‐being pathway. Further evaluation is required to determine whether these perceived benefits translate into measurable improvements in family psychological outcomes.

### 5.2 Recommendations and Clinical Implications

The findings highlight the importance of recognising family members as individuals requiring support throughout the ICU trajectory. Participants described information needs, communication barriers, uncertainty and emotional distress as key sources of burden, suggesting that family support should extend beyond the provision of clinical updates to include structured psychological and emotional support.

The booklet was perceived as an acceptable and accessible resource that may support family‐centred ICU care by improving orientation, facilitating communication with healthcare professionals, preparing relatives for transitions and validating the emotional impact of critical illness. Incorporating such resources into routine ICU pathways may provide families with consistent access to support at a time when distress and cognitive overload may limit their ability to process information.

Nurses were identified as central facilitators of communication and support, highlighting their potential role in introducing and reinforcing family well‐being resources. Embedding the booklet within existing multidisciplinary approaches, including ICU nursing and psychological services, may strengthen continuity of support from admission through recovery and discharge.

Further research should evaluate the effectiveness of the booklet in larger and more diverse populations, including its impact on caregiver psychological outcomes, information needs and engagement with healthcare services. Future implementation studies should explore digital delivery formats, cultural adaptations and integration across different critical care settings.

## 6 Conclusion

Family members perceived the booklet as accessible, acceptable and psychologically supportive. Beyond improving access to information, the resource appeared to provide emotional containment, support communication, validate distress and enhance families' sense of orientation and agency during ICU admission. These findings emphasise the need for family‐centred approaches that recognise relatives as active participants in critical care recovery. The booklet represents a promising component of wider ICU well‐being provision, although further research is required to establish its clinical effectiveness and implementation impact.

## Funding

The authors have nothing to report.

## Conflicts of Interest

The authors declare no conflicts of interest.

## Supporting information


**Data S1:** Family and caregiver ICU support guide and recovery booklet.

**Supporting Information: S1** Table of themes, subthemes and quotes of family members feedback on the booklet.

## Data Availability

The data that supports the findings of this study are available in the Supporting Information of this article.
