# Supplementary material for: The Development, Acceptability and Suitability of an Information and Well‐Being Booklet for Family Members of Intensive Care Unit Patients
Source: Nurs Crit Care. 2026 Jul 20;31(4):e70589. doi: 10.1111/nicc.70589 (PMC13385495; doi:10.1111/nicc.70589)
Supplement: Supplementary file 1 — Data S1: Family and caregiver ICU support guide and recovery booklet. [file NICC-31-0-s001.pdf]

NHS
North East London
NHS Foundation Trust

NHS
Barking, Havering and Redbridge
University Hospitals
NHS Trust

Family and Caregiver ICU Support Guide and Recovery Booklet

Understanding and Coping with a Loved One's Intensive Care Stay

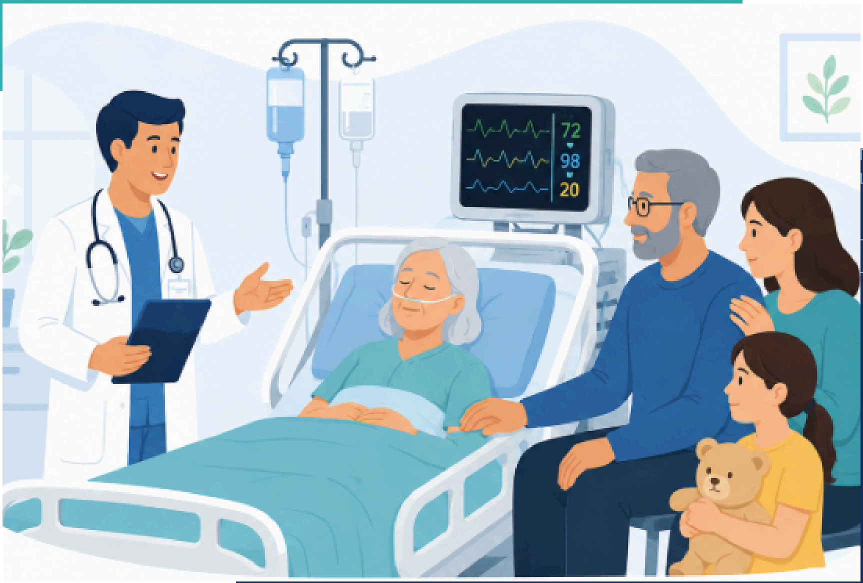

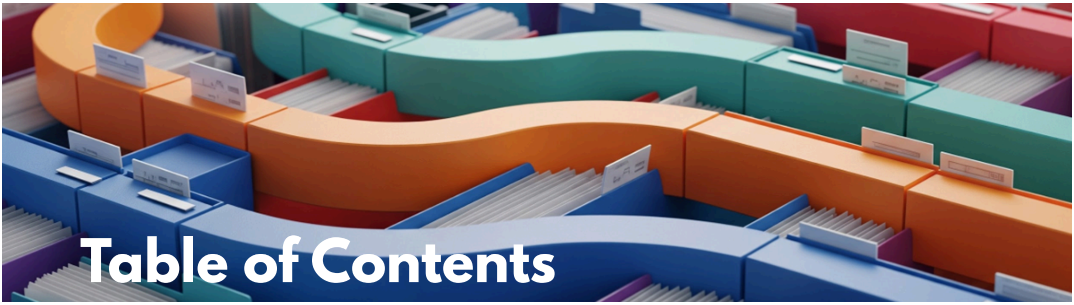

Table of Contents

- **Introduction**
- **The Intensive Care Unit (ICU)**
- **Equipment in ICU**
  - Monitoring Systems
  - Ventilator (breathing machine)
  - Tracheostomy
  - Speaking Valve
  - Drips
  - Nasogastric (NG) Tube
  - Urinary Catheter
- **Key People in ICU**
  - Doctors
  - Nurses
  - Physiotherapists
  - Dieticians
  - Speech and Language Therapists
  - Psychologists
- **Communication**
  - Who you can talk to
  - How you can communicate with the ICU team
  - Interpreter use
- **Delirium**
  - What is delirium?
  - What causes delirium?
  - How long does delirium last?
  - What can you do?
- **Leaving ICU**
  - What to Expect on the Ward
  - Who Can Help on Other Wards?
  - Moving to Another Hospital

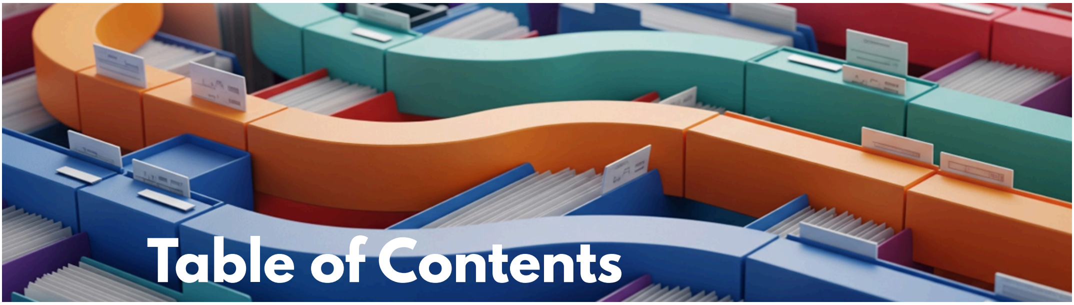

Table of Contents

- **Going Home**
  - What Does Recovery Look Like?
  - What to Expect During Recovery?
    - Physical Symptoms
    - Psychological Symptoms
  - How You Can Help?
  - ICU Follow-up Clinic
- **End of Life**
  - How do we know someone is at the end of their life?
  - Supporting Your Loved One at the End of Life
  - You can look after yourself
  - Grief
  - Support services
- **Wellbeing Tips & Strategies**
  - DURING your loved one's ICU stay
    - Managing other people
    - Visitation
    - Sleep
  - AFTER your loved one's ICU stay
    - Validating your emotions
    - Time management
    - Worry and anxiety
    - Low mood
    - Stress and Memories
- **Support & Help Directory**
  - Carer-specific support
  - Emotional and Mental Health Support
  - Crisis Support
  - Other general support

Introduction

Your relative or loved one is currently or has been in intensive care. Someone may be in intensive care because of an accident, an illness or for treatment after having a major operation. Although this is a difficult time for your loved one, we also know how difficult this time can be for you.

This booklet is designed to provide you with some information about intensive care and what to expect. It also provides some tips and strategies to support your wellbeing whilst your loved one is in intensive care and after they leave hospital.

Everyone's situation is different. Each person and family will have their own needs and their own unique journey. Please feel free to go to the section that feels most relevant to you, depending on where you and your loved one are at in your journey.

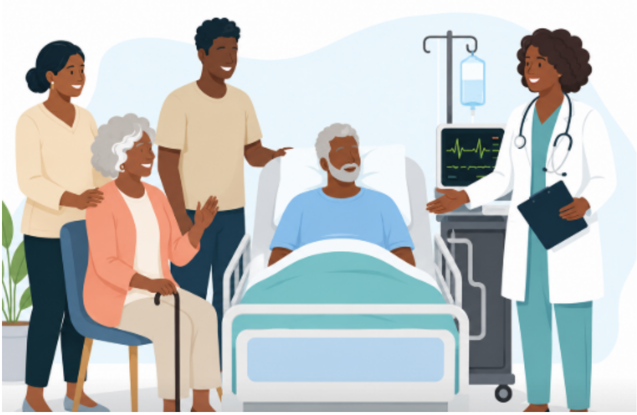

The Intensive Care Unit (ICU)

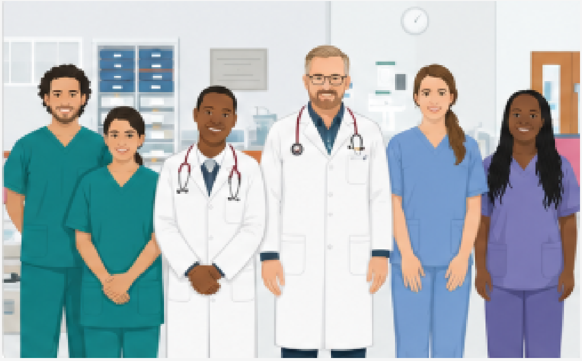

Seeing your loved one in the ICU for the first time can be overwhelming and distressing. They may be connected to lots of machines, wires and drips, and might look very different from how they normally look. They could also be receiving strong pain relief or sedatives, which might make them drowsy, partially conscious, or even unconscious.

If you have any questions about the care they are receiving, please don't hesitate to ask. The ICU staff are here to support you and will be happy to explain what is happening and keep you updated as your loved one's condition changes.

We understand that the equipment, medical terms, and the many healthcare professionals you'll meet can feel confusing. In the following sections, we'll guide you through some of the most common machines, treatments, and the different members of the ICU team who are caring for your loved one.

ICU at Queens Hospital

At Queens Hospital, there are a few different ICUs. So, first, we thought it would be helpful to distinguish between the different ICUs, as we know this can be a little confusing.

General Intensive Treatment Unit
This is the general ICU located in the ground floor blue zone.

Jubilee Ward
This is also a General ICU. This is found on the 4th floor blue zone.

Neuro Critical Care Unit
This ICU is specific for patients with neuro (brain) conditions. Located in the ground floor blue zone.

Equipment in the ICU

When your loved one is in intensive care, you may see a range of medical equipment in use. This can feel overwhelming at first, but each piece plays an important role in supporting their recovery. Your loved one may be supported by one or several of these devices, depending on their condition and needs.

Monitoring System

The monitor is located at the back of the bed space. It gives staff information on patients' vital signs such as blood pressure, heart rate and oxygen levels. It is normal for the numbers to keep changing and alarms may sound. Most of the time this will be caused by a simple reason, such as the patient moving.

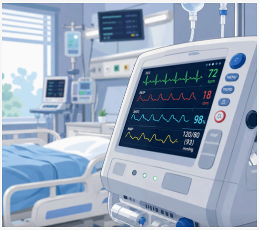

Ventilator (breathing machine)

Some patients in the ICU are not yet strong enough to breathe by themselves. In these cases, they will be connected to a ventilator — a machine that helps with breathing. A ventilator is attached to a thin, soft tube called an endotracheal tube, which is gently placed through the nose or mouth into the windpipe. This tube is connected to the ventilator machine, which moves air and oxygen in and out of the lungs. The ventilator can do all the breathing for the patient, or it can assist their own breathing efforts. As your loved one's condition improves and they become stronger, the support from the ventilator will gradually be reduced. This process is called weaning. Weaning can take different amounts of time depending on the patient — from a few hours or days to several weeks, and sometimes even months. The ICU team will carefully monitor and support your loved one throughout this process.

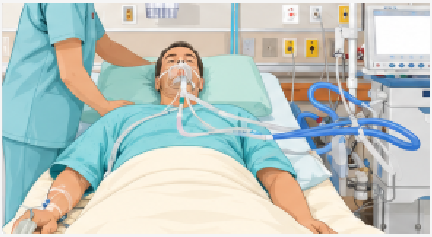

Equipment in the ICU

Tracheostomy

This procedure involves an operation to create a hole in the skin of the throat, called a stoma. A tube is then inserted through this hole, which assists patients with their breathing. This procedure often occurs when patients are likely to remain on a ventilator for more than a few days.

Although this may look strange, it is actually more comfortable for the patient than breathing through a ventilator. Once the tracheostomy is no longer needed and the patient can breathe completely on their own, a doctor will take the tube out and let the hole heal (usually takes around 7 to 10 days).

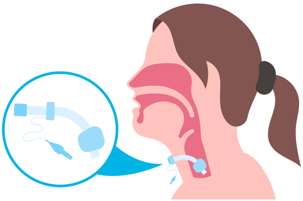

Speaking Valve

When someone has a tracheostomy or breathing tube, they are often unable to speak because air no longer passes through their vocal cords. If it is medically appropriate, a speaking valve may be used. This small device is attached to the tracheostomy tube and allows air to pass through the vocal cords when the patient breathes out. This can enable your loved one to make sounds and eventually speak.

It's important to know that:

- Their voice may sound weak or hoarse at first. This is normal and happens because their vocal cords haven't been used in some time.
- Using the speaking valve can be tiring. At first, they may only tolerate it for a few minutes, but this time will be gradually increased as they build strength.

If you have any questions about this process or how you can support communication with your loved one, ask to speak to a speech and language therapist.

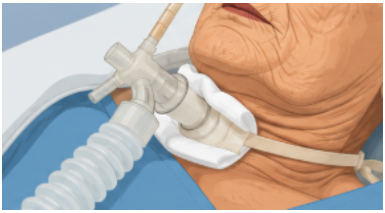

Equipment in the ICU

Drips

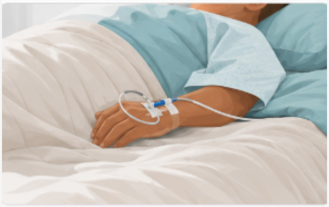

You may notice that your loved one is connected to several drips, also known as intravenous (IV) lines. These are used to give fluids to keep them hydrated, provide liquid nutrition if they are unable to eat, and deliver important medications such as pain relief or medicines to support their blood pressure and other vital functions.

The drips are inserted into a vein, usually in the back of the hand, arm, or sometimes a larger vein in the neck or chest.

Nasogastric (NG) Tube

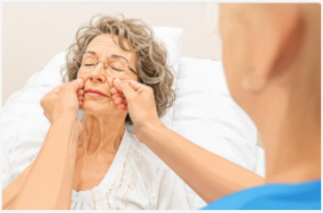

An NG tube is a thin, flexible tube that is gently inserted through the nose and down into the stomach. While it might look uncomfortable, the tube is very flexible and thin, so it generally does not cause any pain.

This tube is often used as a short-term way to provide nutrition when a patient is unable to eat or swallow safely. It can also be used to remove fluid or air from the stomach, depending on the patient's needs.

Urinary Catheter

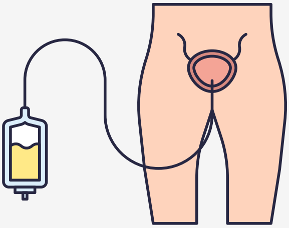

A urinary catheter is a thin, flexible tube that is inserted into the bladder to drain urine. This is used when a patient is unable to pass urine naturally, which can happen when someone is very unwell or sedated.

The catheter allows doctors and nurses to closely monitor how much urine is being produced, which helps them assess how well the kidneys are working and make important decisions about treatment.

If you have any questions about the equipment mentioned above, or anything else you see in the ICU, please don't hesitate to ask a member of staff. We're here to help and will be happy to explain anything further

Key People in ICU

Your loved one is being looked after by a team of dedicated healthcare professionals, all working together to provide the best possible care.

You will encounter many different team members during your loved one's stay; while it might be hard to remember everyone, each person plays an important part in their care. Please feel free to ask staff for their names or to explain their role. We are here to help you feel as informed and supported as possible.

In ICU, nurses, doctors and consultants work in shifts, so the team looking after your loved one may change from day to day. We understand that this can sometimes feel unsettling or anxiety-provoking. Please be reassured that detailed handovers take place between staff to ensure that all relevant information is shared and that care remains consistent and safe.

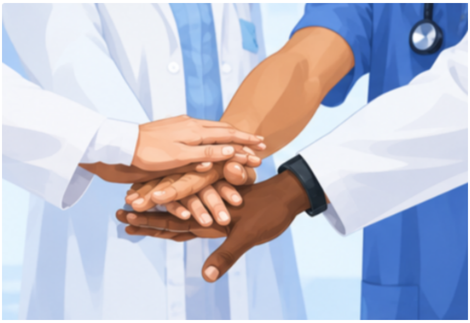

Here's some information about key members of the team:

Key People in ICU

Doctors

A medical consultant leads the ICU medical team which consists of doctors of different grades and is responsible for your loved one's overall treatment plan. They usually visit daily to review progress and make decisions about treatment and care.

Other specialist doctors — such as anaesthetists, orthopaedic surgeons, or other specialties — may also be involved, depending on your loved one's needs. They work closely with the ICU team before, during, and after their ICU stay, helping to ensure a smooth transition when your loved one is ready to move to a general ward or back home.

Nurses

ICU nurses are specifically trained to care for critically ill patients. Each nurse usually looks after just one or two patients at a time, allowing them to give close, focused care. They provide most of the hands-on treatment and continuously monitor the patient's condition. Your loved one will have an assigned nurse each day, and their name should be written on the board above your loved one's bed. If you have questions about your loved one's daily care or progress, their assigned nurse is often the best person to ask first.

Physiotherapists

Physiotherapists play a crucial role in a patient's recovery to help patients regain strength and mobility. They will listen to the patient's chest and help ensure their lungs are kept clear. Even if the patient is asleep, physiotherapists will gently exercise their arms and legs to prevent their muscles and joints from becoming weak or stiff. Once patients get better, physiotherapists will help them with exercises to build up muscle strength to eventually start resuming daily activity again.

Key People in ICU

Dieticians
A dietitian will be involved in planning your loved one's nutritional care. They will assess what your loved one needs and decide the best way to provide nutrition while they are unable to eat normally. This may involve feeding through a nasogastric (NG) tube, which goes through the nose into the stomach. If food can't be safely given into the stomach, nutrition may be provided through a drip directly into a vein. The dietitian will continue to review your loved one's needs throughout their ICU stay to help support their recovery.

Speech and Language Therapists
Speech and language therapists support patients with communication and swallowing, especially if a tracheostomy is in place, which can make speaking difficult. They may help your loved one find ways to communicate, such as using a speaking valve on the tracheostomy if it's safe to do so. They also assess swallowing to ensure it's safe to start eating and drinking again when the time is right.

Psychologists
The ICU Psychology Team is here to support not only your loved one but also you, through the emotional challenges of being in ICU. They can offer a confidential space for you to talk about your feelings, whether that's anxiety, sadness, trauma, stress, low mood or feeling overwhelmed. If you feel you or your loved one would benefit from talking to someone, please don't hesitate to ask the nurse looking after your loved one to contact the ICU Psychology Team for you. We see patients and carers when they are in ICU or on the ward, and can also see people after they have left hospital.

Communication

We understand how important it is for you to stay updated about your loved one's progress and to feel connected to the team caring for them. Clear communication is a priority for us and the following section explains the best way to get in touch.

To help our team from repeating the same information to different family members or friends, we recommend that your family nominate one person to be the main point of contact. This person can then share updates with other relatives and friends.

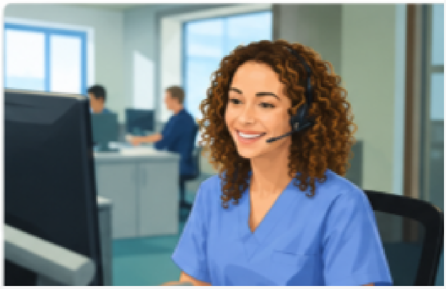

How to communicate with the ICU team
The most direct way for the nominated spokesperson to get in touch is by telephone. When you call, the ward clerk or a staff member will answer and can direct your call to the appropriate nurse. Please be aware that the top priority of our nurses is direct patient care, so if they are in the middle of a task, they may need to arrange a time to call you back.

To help our team from repeating the same information to different family members or friends, we recommend that your family nominate one person to be the main point of contact. This person can then share updates with other relatives and friends.

Who You Can Talk To

Assigned Nurse

Your loved one's assigned nurse is usually the best person to speak with for daily updates. As they provide direct, hands-on care, they will have the most current information about their condition and treatment plan.

Doctor in Charge

A Doctor is responsible for overseeing your loved one's overall treatment plan while they are in ICU. Doctors usually visit each day to assess progress, adjust treatment, and make important decisions about care. If you have specific questions about your loved one's overall condition, treatment plan, or progress, these are best directed to the Doctor in charge.

Nurse in Charge

Each shift will have a senior nurse to oversee the entire unit. If the assigned nurse of your loved one is unavailable, or if you have a more general concern you would like to discuss, you can always speak with the nurse in charge. They are usually found at the front desk in the unit.

Psychology Team

We recognise that this is a stressful and challenging time for you. Our psychology team is available to provide support for families and carers. If you are feeling overwhelmed or would simply like someone to talk to, please don't hesitate to reach out.

It can sometimes feel difficult to ask, especially during such a stressful time, but your questions are important. Please feel able to ask at any time — the team is here to support you.

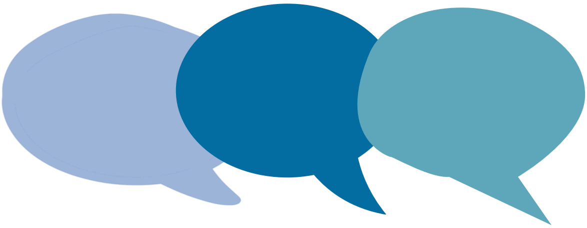

Interpreter Use

Even if you speak English, but feel more comfortable speaking in your mother tongue, we will make every effort to support that. Feeling heard, understood, and at ease is very important to us, and we want the ICU experience to feel as familiar and respectful as possible for you and your family.

If English Is Not Your First Language: Interpreter Use
We know that many of the people we care for in ICU - and their families and friends - speak languages other than English.
Clear communication is essential, especially when it comes to understanding your loved one's condition, treatment, and recovery.
We want you to feel fully informed, involved, and supported every step of the way.

If you, your loved one, or anyone else involved in their care would prefer to speak in a language other than English, please let a member of staff know - this could be the assigned nurse, nurse in charge, or doctor. We will do our best to arrange for a professional interpreter to help facilitate these important conversations.

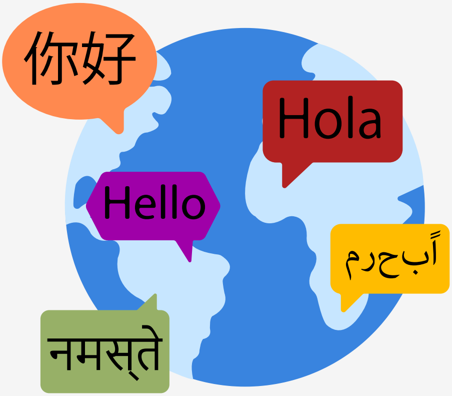

Delirium

What is delirium?
Delirium is a medical condition that causes sudden and severe confusion. It's often described as feeling like a nightmare, but feels completely real to the person experiencing it. People with delirium may have hallucinations — seeing, hearing, or feeling things that aren't actually there. They can become disoriented and believe they are in entirely different situations, many of which can be distressing or frightening.

For example, a person with delirium might:

- Not realise they are in a hospital
- Believe they see dangerous people/animals nearby
- Think they have been kidnapped
- Mistake healthcare staff for imposters

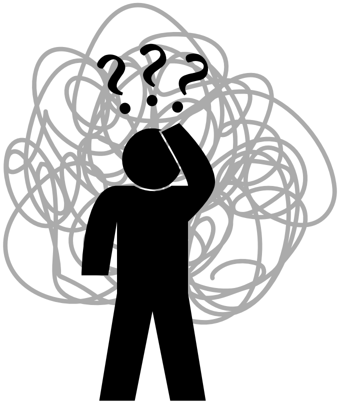

Believe they or their loved ones have died

Delirium can be really scary, both for the person experiencing it and for those around them.

What causes delirium?
When someone is very unwell in ICU, it is common for people to become confused. This confusion is unfortunately very common (approximately 80% of ventilated patients in ICU) and can happen for many different reasons.

Delirium

Typically, reasons why someone might develop delirium include:

- Infection
- The drugs given to patients to help treat their illness or condition
- Kidney, heart or lung difficulties/failure
- Some intensive care patients are more likely to get delirium, such as:
- Older patients or those who had become forgetful before their ICU treatment
- Those who were already on medication before ICU treatment
- Those who have liver problems or patients on ventilators — at least two out of every three ventilated patients will experience delirium

How long does delirium last?
It is usually temporary and will last from a few days to a week. Sometimes, it can last longer and may take several weeks or months to resolve.

Because the person is in and out of consciousness during delirium, they are likely to have what we call fragmented memories of their experience and may feel quite confused about what happened. Sometimes people can feel like they are "losing their mind". You can help them make sense of it by explaining that they have experienced delirium and that this can affect memory, causing confusion about what was real and what was not. This can reassure them that what they are experiencing is a common and a temporary part of recovery.

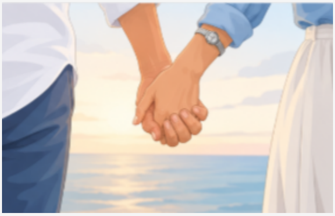

Delirium

What can you do?
If your loved one is experiencing delirium, there are many ways you can support them. The most important thing is to offer reassurance and talk to them. Simply hearing a familiar, comforting voice can make a huge difference. Here are some helpful ways to support them:

- Holding their hand, and reassuring them.
- Use simple words and sentences.
- Orientation: telling them often where they are, what day it is, what the time is and that they are safe.
- Talking with them. If your loved one is sedated, try reading a favourite book or a newspaper to them, or just talk to them about what is going on in your life.
- Hallucinations and delusions are a common symptom of delirium. Try not to dismiss what they are experiencing. Instead, try to respond to the emotion underlying their experience, and emphasise that they are in a safe place.
- Bringing in familiar objects, for example a blanket, photos, pictures, and place them around the bedside where your loved one can see them.
- You might also find a whiteboard ('get to know me board') by your loved one's bed. Fill this in with their likes, dislikes, pictures and anything else.

ICU diary: Your loved one will likely have confused and gap-filled memories about their time in ICU. Keeping a diary for them and noting down key things that have happened each day can be really helpful. We provide these diaries for you, so the nurses will be able to get you one if you ask.

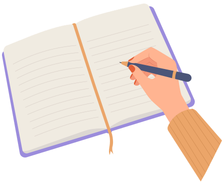

Leaving the ICU

It's completely normal to feel anxious or unsure when your loved one is moved from the Intensive Care Unit (ICU) to the next destination. As a carer, you've likely become familiar with the ICU team and routine, and it's understandable to feel uncertain about what comes next.

What to Expect on the Ward

You will notice that once your loved one gets to the ward, there are fewer nurses for each patient compared to ICU. This is because your loved one is getting better and is more independent so requires less one-to-one care than they needed when they were in ICU.

This change can feel like a big adjustment — for both of you. It is not uncommon for patients to experience changes in mood or feelings of loneliness during this transition, even as they continue to recover physically. If you notice any emotional changes or feel concerned about how they're coping, please speak to the nurse in charge. You can also refer to the wellbeing tips and strategies section of this booklet.

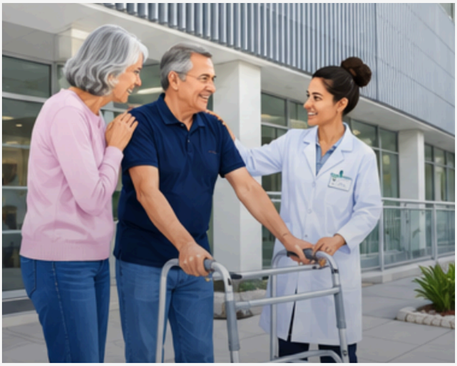

Leaving the ICU

Who can help on other wards?
The nurses, doctors and physiotherapists working on the wards are there to help, advise and support you. If you are worried about anything, discuss it with them. If you have questions about your loved one's stay in ICU, they will be able to contact the ICU staff for you if necessary.

The ICU Psychologists may also see you on the ward to ensure a smooth transition and to address any concerns or psychological distress resulting from you or your loved one's experience in ICU.

Occupational Therapists
As your loved one begins to recover, you may meet Occupational Therapists (OTs). They play a key role in supporting recovery and helping patients regain their independence. OTs assist with everyday activities such as sitting up, washing, dressing, and other tasks that are essential for daily life.

Moving to Another Hospital
If your loved one needs specialist care that is unavailable at this hospital, a transfer to a critical care unit or specialist ward at another hospital may be recommended. A trained team of staff will oversee the transfer, using stabilised equipment and ventilatory support suited for travel. Once a transfer decision is made, the medical and nursing team will let you know why and where your loved one is being moved, when it will happen, and contact information for the receiving team.

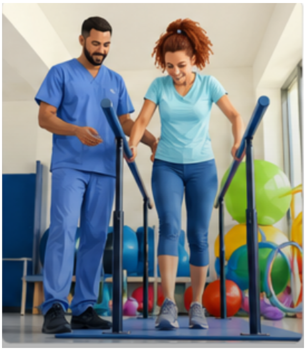

Going Home

Going home is a significant step in yours and your loved one's journey. You will probably be pleased that your loved one is able to go home. However, you might also feel worried about how you or your loved one will manage.

When someone has been critically ill, recovery is often a long process, even after leaving hospital. Exactly how long this will take will depend on the length of time your loved one has been ill, how much their illness has affected their body (for example if they have lost a lot of weight or muscle strength that they will need to rebuild gradually) and if there are any other physical health complications that require rehabilitation or further treatment.

Recovery is a journey. Some days may feel like one step forward and two steps back, but every day is a milestone. It's important to celebrate small improvements and recognize that ups and downs are a normal part of the recovery journey.

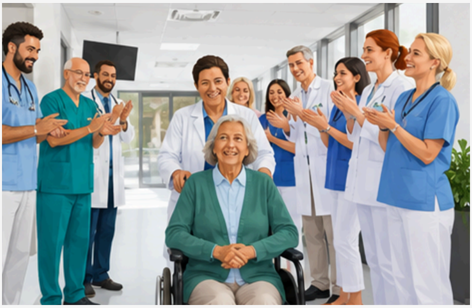

Supporting a loved one after an ICU stay: What does recovery look like?

Following your loved one's ICU stay, they will need time to recover both physically and psychologically. While it's natural to feel concerned if their progress seems slow, it's important to remember that everyone heals at their own pace. For many, recovery can take weeks or even months to fully recover.

Not all patients will return home immediately after leaving the hospital. Some may be transferred to a specialist rehabilitation centre for additional care before going home. Others may go directly home but receive support from community services.

If community support is arranged, you may be contacted by professionals such as Physiotherapists, Occupational Therapists, Speech and Language Therapists, or other members of a specialist rehabilitation team, depending on your loved one's needs.

Before your loved one is discharged, you will be informed whether any follow-up care or support services have been arranged. Some patients, particularly those who have recovered well in hospital, may not require any ongoing care at home.

If you have any concerns about your loved one's recovery after discharge, please don't hesitate to raise them during the Follow-Up Clinic.

What to Expect During Recovery

What to Expect During Recovery
Many people experience a range of symptoms after leaving the ICU. These can affect their ability to return to everyday life and may feel overwhelming at times. Here are some common challenges your loved one might face:

Physical symptoms:

- Shortness of breath, even with mild activity
- Muscle weakness and difficulty moving around
- Ongoing fatigue
- Reduced appetite
- A hoarse or weak voice

Psychological symptoms:

- Feeling anxious, nervous, or low in mood
- Difficulty sleeping or concentrating
- Nightmares, flashbacks or confusing memories from their ICU stay

What to Expect During Recovery

These symptoms can impact your loved one's ability to carry out routine activities and may make them feel frustrated, discouraged, or even defeated. It's important to acknowledge these feelings and reassure them that they are not alone.

How You Can Help

As a family member or loved one, your presence and support can make a huge difference. Simply being there — listening without judgment, offering encouragement, and helping with small tasks — can be comforting.

Here are a few tips to support their recovery:

- Be patient: Progress may be slow and uneven.
- Encourage independence where possible: Let them try things on their own while being available if needed.
- Talk openly: Let them share their feelings and experiences without rushing to fix things.
- Celebrate small wins: Every step forward, no matter how small, is progress.

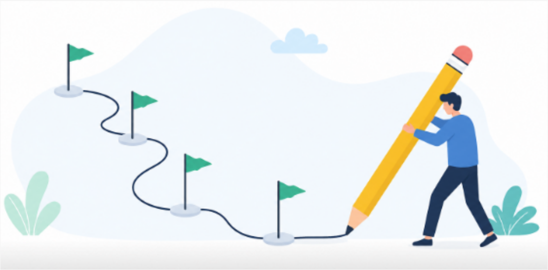

ICU Follow-Up-Clinic

ICU Follow-Up Clinic

If your loved one was admitted to the ICU for more than three days, you will receive a letter approximately three to six months after their discharge, inviting them to attend an ICU follow-up clinic. The clinic is run by a multidisciplinary team, with representatives from each professional group involved in your loved one's ICU care.

This clinic provides support and guidance for any physical or psychological challenges your loved one may be facing as a result of their ICU stay. It's an opportunity for us to check on their recovery progress and ensure they are receiving the right support. The follow-up clinic also allows both you and your loved one to talk about your experience and ask any questions you may have related to their ICU stay.

While the clinic focuses on your loved one's recovery, we also want to know how you are coping. If you're finding things difficult or have any concerns about your own wellbeing since your loved one's ICU admission, please let the team know. We're here to support you too.

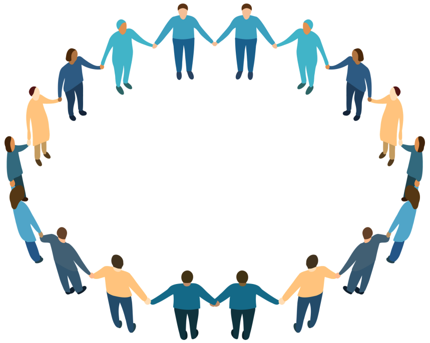

End of life: If your loved one does not survive

Intensive care treatment can support people when they are very ill. However, despite the best efforts of the ICU team, sometimes a person may be too unwell to recover. If their condition isn't improving and continuing treatment would not be in their best interest, the care team will discuss the next steps with you and explain how they will provide care during their final stages of life.

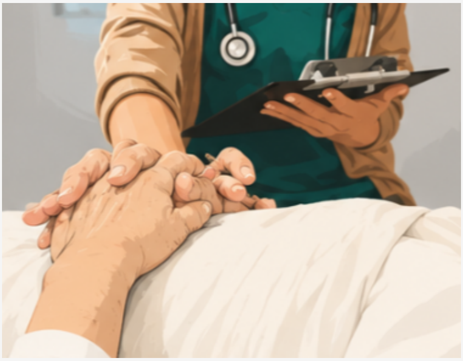

How does the team know if someone is at the end of their life?
As discussed above in the equipment section, ICU treatment uses machines and medicine to help people when they are critically ill. Patients may only be partly awake or they may have been given strong drugs to keep them asleep (induced coma). This might make it difficult for you to see that your loved one is getting more ill.

The ICU team will be constantly monitoring your loved one to see how they are doing. They will be checking all of the machines attached to your loved one, as well as other indicators like blood test results, heart rate and blood pressure. All this information makes it possible for the team to determine whether your loved one is getting better, staying the same, or getting worse.

End of Life

What you can do to support your loved one at the end of their life

Many families find comfort in being able to do small, gentle things for the person they love. Please speak to the care team looking after your loved one to see if there are ways you can support them in their final days or hours.

There are many ways to offer comfort at the end of life:

- **Simply be there:** The most important thing you can offer is your presence. Just being with your loved one, holding their hand, and speaking to them in a calm and reassuring voice can be a profound source of comfort. You might consider reading to them, playing their favourite music softly, or simply just sitting and being with them.
- **Help with gentle care:** With the help of the nurse, you may wish to help with simple acts of care. This could include moistening their lips, gently brushing their hair, or massaging their hands and feet.
- **Connecting with others:** If there are other important family members or friends who are unable to visit, you may want to arrange a phone or video call so your loved one can hear their voices.
- **Spiritual and religious beliefs:** Please let us know if your loved one has any spiritual, religious or cultural beliefs that are important to them, including things they would like to happen before or after their death.
- **Personal items:** You are welcome to bring in small, meaningful items like photos that are important to your loved one.
- **Bedspace adjustment:** Let us know about changes that could be made to the bedspace, such as lowering lighting, or playing music which they like.

End of Life

How you can look after yourself after a loss

It is important that you take care of yourself during the hours and days after your loved one's death. The following suggestions are about the immediate sources of practical and emotional support available to you and your family:

- **Seek help from your network:** Do not be afraid to accept help from friends and wider family. Allow them to support you with practical things like meals, phone calls and household tasks so you can have the space you need.
- **Appoint a communicator:** You might find it helpful to ask one person to be the main point of contact to share news and general arrangements with your wider circle.
- **Use the support available here:** The chaplaincy and pastoral care team continues to be available to support you. The bereavement services office can also provide practical advice and guide you through the immediate next steps, such as death registration and other arrangements.
    - Queen's Hospital bereavement support
    - King George Hospital bereavement support

- **Talk with the medical team if needed:** You may have more questions about your loved one's illness. We can often arrange a follow-up meeting with a member of the ICU team to talk through what happened.
- Refer to help directory for additional support available to you.

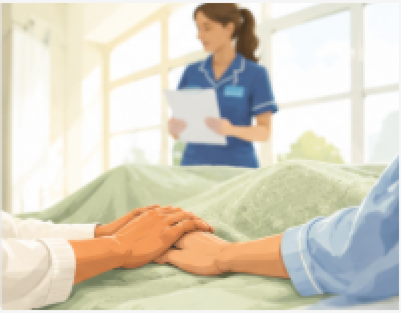

Grief

Losing someone you love can leave you with complex feelings. You may experience a range of emotions like devastation, anger, numbness, and sadness, together with physical symptoms like tiredness and exhaustion. Grief is a natural response to death, it is deeply personal and there is no 'right' or 'wrong' way to feel. When a death happens in the ICU, it can be particularly shocking and can lead to strong emotions, especially when the death was unexpected.

Sometimes, families may experience:

- **Numbness and nothingness:** To begin with, you may feel numb or detached. What happened may seem unreal. This is a common way our minds cope with overwhelming shock.
- **Relief and guilt:** You may feel a sense of relief that your loved one's suffering has ended. At the same time, this relief can be accompanied by feelings of guilt. Although this might feel confusing this is a natural part of the grieving process.
- **Difficult memories:** You may experience strong, intrusive memories or images, sometimes called 'flashbacks'. This is also a normal, though distressing, part of processing a traumatic event.

The grieving process can affect both your body and your mind. You may find it difficult to sleep, lose your appetite, or feel more anxious or irritable than usual. It is important to find support to help you cope. Here are some support services and resources that may be of help:

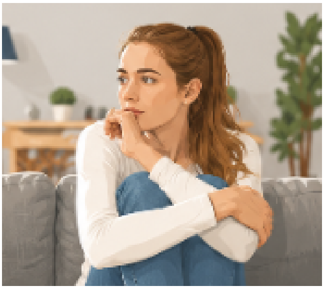

Support Services

King George Hospital
Bereavement Support: https://www.rns.uk/king-george-hospital-83317
Chaplaincy Office:
Extension: 8494
External Tel: 020 8970 8494
Email: Bhrut.chaplaincy@nhs.net

Queen's Hospital
Bereavement Support: https://www.rns.uk/queens-hospital-romford-72721
Chaplaincy Office:
Extension: 4729/3201
External Tel: 01708 504329/503201
Internal Staff DECT phone: 6255
Email: Bhrut.chaplaincy@nhs.net

Cruse Bereavement Support
Free Helpline: 0808 808 1677
Email Service: https://www.cruse.org.uk/postcode-lookup/
Opening times:
Monday, Wednesday, Thursday, Friday – 9.30am – 5pm
Tuesday – 1pm – 8pm
Saturday & Sunday – closed

Child Bereavement UK
Email: helpline@childbereavementuk.org
Telephone: 0800 02 888 40
Live Chat: visit https://www.childbereavementuk.org/

Mind UK
For more bereavement resources, visit Mind

While it may feel difficult, taking small steps to care for your physical and emotional well-being can help you navigate the days ahead. Please refer to the "Wellness Tips and Strategies" section for a more detailed explanation.

Supporting Children & Young People

In addition to caring for your loved one in the ICU, you may also have the added responsibility of looking after young children. Along with the many worries you may have, it can be hard to know the best way to help the children in your family. Below is some information that may help you to support a child in this situation:

- Explain to the child what is happening in a straightforward and simple way (e.g. "Daddy had bad headaches, which made him very poorly. We took daddy to hospital and the doctors said the headaches were a sign of a bigger illness in his head. The doctors and nurses are doing everything they can to help him"). What you tell the child will depend on their age and why their relative was taken into the ICU, but it is very important that you do explain what is happening. Not knowing is likely to make them feel anxious or confused.

- Let children know it's okay to ask questions. This helps them take the lead in finding out what they want to know. Often, younger children may ask more freely, while older children and teenagers can be more reserved or hesitant. Whatever their age, it's important to reassure them that their questions are welcome — and that they can talk to you, or to other trusted family members or friends, whenever they need to.

- It's important to make sure children have emotional support during this time. Friends, family, and schools can play a key role. You may want to inform their teachers or trusted adults about the current situation, so they can provide extra understanding and support.

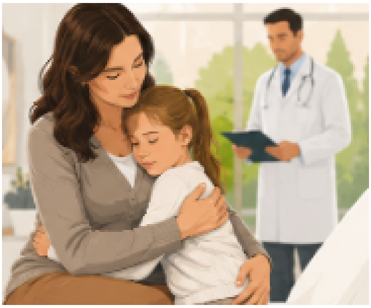

Help Directory

Here are some services and resources you may find helpful and who might be able to support you or your child further if necessary:

Winston's Wish
A charity for bereaved children, who produced an information book As Big As It Gets: Supporting a child when a parent is seriously ill. This includes information and activities, sources of information and support and recommendations of books that may help children during this time.
Helpline: 08452 03 04 05
Website: www.winstonswish.org.uk

Family Lives
A national family support charity providing help and support in all aspects of family life.
Helpline: 0808 800 2222
Website: www.familylives.org.uk

ChildLine
A free helpline for children and young people in the UK, and they can call to talk about any problem that they have.
Free helpline: 0800 1111
Open 24 hours a day, 7 days a week.

Wellbeing Tips & Strategies

DURING your loved one's ICU stay

You can help your loved one by looking after yourself. You should not feel guilty for not being by their bedside 24 hours per day. Feeling tired, stressed, or even frustrated is normal — caring for someone in ICU is emotionally and physically exhausting. It is important that you give yourself a break.

Your loved one will be very well cared for and the staff will contact you straight away if they need to or if there is any change in their condition. Here are some general tips to help you through this difficult time:

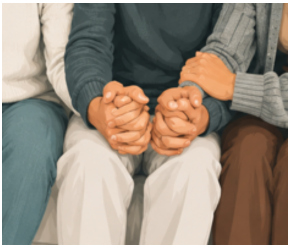

Managing other people

Your family and friends may be worried about you and your loved one, and they might want updates on how things are going. While you may appreciate their concern, it can become exhausting if the phone keeps ringing nonstop.

It might be easier to share updates via email or text with multiple people at once. Alternatively, you could designate one person to receive regular updates and have them share the information with everyone else.

Visitation

Visiting hours in all of the ICUs is between 14:00 and 19:00. Patients are allowed two visitors at their bedside at a time, taking turns if necessary. This is for the comfort and safety of other patients in the same area.

If your loved one moves to a general adult ward, visitors here are welcome from 11.00 to 19.00.

Wellbeing Tips & Strategies

Sleep

Your sleep may have been disrupted. The following tips might help you adopt a good sleeping pattern:

- Set an alarm to wake up at the same time every day (even at the weekend): This anchors your body clock and makes it more likely that you will feel sleepy at the same time each night.
- Try not to spend any time in the bed or bedroom during the day: Spending time in the bedroom when awake creates an association between the bedroom and wakefulness. If you need to lie down during the day, try to do so on a sofa or somewhere else outside of the bedroom.
- Avoid napping in the day: Any sleep you have during the day will make it harder to sleep that night. If you are feeling very tired during the day, bright light, fresh air, interactive activities and gentle exercise will help you stay awake.
- Try to have at least an hour of wind-down time before bed: This should be a time to relax when you don't have to do any chores or work.
- Only go to bed at night when you feel sleepy: You should be struggling to keep your eyes open before you get into bed.
- If you find you are awake at night and can't get back to sleep, it is better to get out of bed and do something relaxing and enjoyable rather than lying in bed worrying or feeling frustrated. Go back to bed only when you are struggling to keep your eyes open.

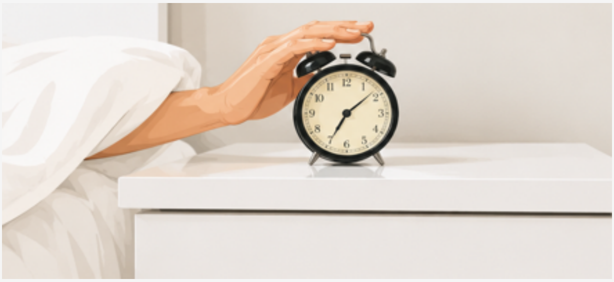

Wellbeing Tips & Strategies

AFTER your loved one's ICU stay

Although leaving hospital and the prospect of recovery can be difficult for your loved one, we also know this can be a really difficult time for you too. Caring for someone can take a heavy physical and emotional toll, and processing your own experiences during their time in the ICU can add to the strain. It is therefore really important that you look after your own wellbeing too. Below is a list of wellbeing tips and strategies that may help support you through this time:

Validating your emotions

We know that caring for someone after an ICU admission can result in many different emotions, including exhaustion, guilt, resentment, worry, anxiety, anger and more. This is normal. These emotions are a natural response to a stressful situation.

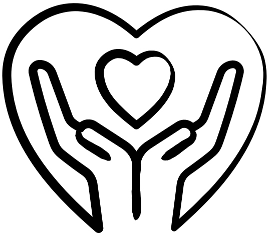

Time management

After spending a lot of time in ICU caring for your loved one, you may have responsibilities built up from being unable to attend to them whilst your loved one was in ICU. To avoid feeling overwhelmed during these times, it is important that you manage your time well. The following tips may help you with this and ensure you are able to complete your tasks with minimal stress:

Wellbeing Tips & Strategies

- Being organised: As a carer, you may be balancing many responsibilities. Writing things down can help you stay on top of tasks like appointments, medications, and daily to-dos. Using the notes app on your phone or keeping a dedicated notebook for to-do lists can make a big difference in staying on top of things and reducing stress.
- Prioritise your tasks: Identifying which tasks are most urgent can help you focus on what needs to be done first. By tackling the most important tasks early, you ensure that the essentials are taken care of — even when time is limited.
- Create a schedule: Setting aside specific times for tasks can help you build a routine. For example, you might tackle urgent tasks in the morning when your energy is highest, and leave less demanding tasks for later in the day.

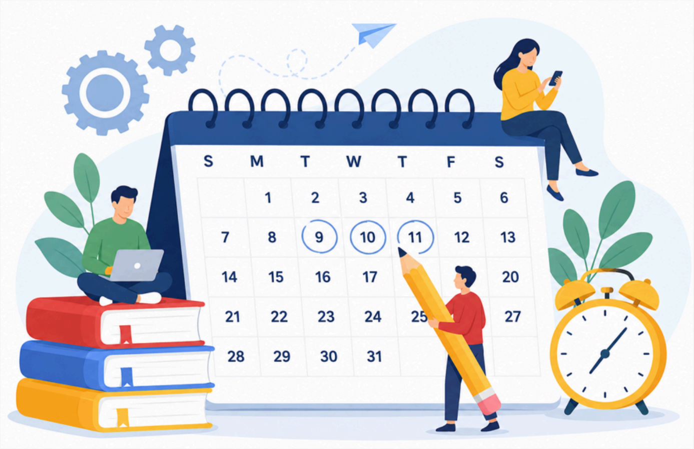

Wellbeing Tips & Strategies

Worry and anxiety

Everyone feels worried or anxious at times. Anxiety is a healthy reaction to stress. You might worry about your loved one's health or that something bad might happen to them. You might also worry about what the future looks like for yourself or your loved one. You might experience tense muscles, faster breathing or sweatiness. You might also feel like you are unable to control your worries. While low levels of stress is healthy, high levels may not feel good, so here are some strategies to help you deal with worry or anxiety:

- **Relax:** Practicing relaxation strategies regularly can help calm and soothe your mind and body.
- **Distraction:** We can worry or do something else...not both. Try to engage your mind in mental activity (e.g. a crossword or Sudoku).
- **Exercise:** Physical activity also occupies the mind – if there is a time in the day you are always more worried, plan activity just before that time.
- **Socialising:** Surrounding yourself with friends or family and engaging in social activities (e.g. hiking, joining carer support groups) can significantly reduce your anxiety and can also act as a temporary distraction from your worries.
- **A worry period:** To stop yourself from worrying all day, set aside a time and place (not in bed) during the day when you deliberately think of all your worries for 15 minutes. Try noticing moments when you start worrying outside of this time, and postpone these worries to the worry period.

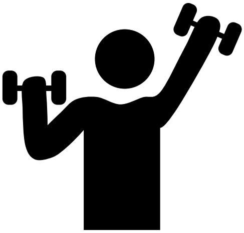

Wellbeing Tips & Strategies

Low mood

Given everything that you have been through, it is natural to feel low in mood. You may feel tearful or sad, or uninterested in activities you normally enjoy. Try to be kind to yourself and remind yourself that you have been through a lot.

How to deal with feeling low:

- **Be active:** Research shows that physical exercise improves mood and general mental health. Any exercise is better than nothing, and even a 10-minute walk can clear your mind and be good for your wellbeing!
- **Talk to someone you trust:** It is usually a relief to be able to express your feelings to someone you are close to. Do not feel ashamed of feeling low, there is nothing wrong with you. This is a normal reaction to stress.
- **Join a carer support group:** By talking to other carers in similar situations as yourself, you may find relief in sharing your experiences. Not only this, but hearing others' experiences may also comfort you in knowing that you're not alone.
- **Activity planning:** When people are low in mood, they often become less and less active, and this makes them feel even worse. To tackle this, you can plan daily activities for yourself using an activity chart. Do not only plan jobs that you have to do. You should also plan activities that you normally enjoy doing. Even a quick coffee or a walk with a friend could be beneficial for your health.

However, if none of these methods work, you may find it beneficial to talk to a trained professional (e.g. GP, psychologist). While it may be natural to have some feelings of low mood, if you experience continuous low mood or feel depressed for more than two weeks, we recommend that you see your GP for further support.

Stress and Memories

You may have upsetting memories about your time in ICU. These memories can be vivid and can come to mind when you don't want them to. This type of memory is called a "flashback" and is caused by stress.

Other symptoms of stress include:

- Irritability
- Loss of concentration
- Anxiety
- Memory lapses
- Low mood
- Being withdrawn
- Having disturbed sleep
- Being jumpy
- Feeling that things will go wrong

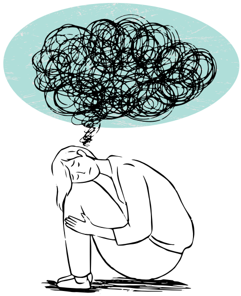

These symptoms are normal after a stressful experience and in most cases will improve within a few weeks.

If these symptoms persist for longer it can be a sign of post-traumatic stress disorder. In this case it is a good idea to seek professional help. Your GP can refer you to an appropriate service to help you.

Wellbeing Tips & Strategies

If you are waiting for professional help, or you decide you do not need professional help, the following ideas for coping with post-traumatic stress have been suggested by experts. Try them out and see what works for you:

How to deal with flashbacks:

- Concentrate on things around you: colours, smells, objects, to bring you back to 'now'. Describe their details to yourself.
- Find a safe smell (e.g. perfume or aftershave) or personal object that you can carry and squeeze, when you need to come back to the present.
- Use a 'grounding statement' that reminds you that you are not in critical care now and that keeps you in the present. For example, "I am now home, I am safe".
- Rehearse an image in your mind that makes you feel safe and in control, which you can bring to mind if a flashback occurs.
- Tell someone you trust how to recognise when you are having flashbacks or nightmares and to use a word or gesture you think will help stop them e.g. remind you where you are, bringing you back to the here and now.

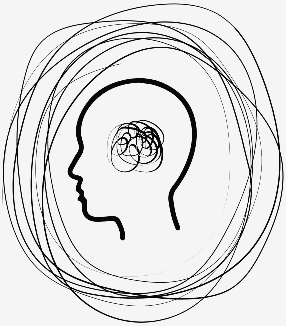

Support & Help Directory

Caring for someone after an ICU stay can be physically and emotionally demanding. Support is available from the moment you need it, and accessing help early can make a real difference.

Carer-specific support

Carers UK
National charity supporting carers with advice, resources, and advocacy.
Phone: 020 7378 4999 | www.carersuk.org

Carers Trust
Provides support, information, and services for carers across the UK. Offers local advice, respite, and practical help.
Phone: 0300 772 9600 | www.carers.org

Headway
Supports people with brain injury and their carers, including advice and rehabilitation resources.
Phone: 0808 800 2244 | www.headway.org.uk

Community and Post-ICU Support

Critical Care Recovery
Guidance and resources for patients and families following ICU.
www.criticalcarerecovery.com

ICUsteps
Charity offering peer support for ICU survivors and their families.
www.icusteps.org

Local NHS community rehab services: Physiotherapy, occupational therapy, and specialist rehab teams may continue care at home. Ask your hospital team for referral information.

Support & Help Directory

Emotional and Mental Health Support
Psychological therapies, also known as 'talking therapies', are free on the NHS and can be accessed from your local community mental health service. You can be referred by your GP or many services accept self-referrals. Check the NHS website to locate your nearest service.

Mind
A national mental health charity which provides advice and support to people experiencing mental health problems. On their website there is lots of useful information on mental health problems such as depression, anxiety and PTSD, including how to access help and support.

You don't have to wait until you feel overwhelmed - accessing support early is important for your wellbeing and your ability to care for your loved one.

Samaritans
Provides confidential, unbiased emotional support, 24 hours a day, for people who feel distressed, desperate or suicidal.
Helpline: 116 123 | www.samaritans.org

Critical Care Support Network
Provides a safe space for families and friends of ICU patients, offering emotional support and practical advice.

How to get help in a crisis or emergency
If you or your loved one feel like you might attempt suicide, or may have seriously harmed yourself, you need urgent medical help. Please:
- Call 999 for an ambulance
- Go straight to A&E, if you can

If you can keep yourself safe for a short while, but you still need urgent advice:
- Contact NHS 111
- Samaritans: 116 123 (24/7)

Support & Help Directory

General Support Services

NHS Services

Call 111 for non-emergency help | www.nhs.uk

Patient Health Information

Explore clear, trusted advice and information from UK doctors and experts.
www.patient.co.uk

Healthtalk

A website on patient experiences of healthcare including intensive care.
www.healthtalk.org

Intensive Care Society

Provides information to help patients and their relatives understand intensive care, what to expect during treatment, and the recovery process, along with practical and emotional support for families.
www.ics.ac.uk

Critical Care Recovery

Information and support to help patients and families understand and manage recovery after intensive care.
www.criticalcarerecovery.com

ICUsteps

A UK charity offering support and information for intensive care patients and their relatives.
www.ICUsteps.org.uk

Mind

Contains information and advice on a wide range of mental health topics.
0208 519 2122 | www.mind.org.uk

British Association for Counselling and Psychotherapy

Information about counselling and psychotherapy, and a directory to find qualified therapists.
01455 883300 | www.bacp.co.uk

Support & Help Directory

British Psychological Society
Provides information about psychology and a directory of qualified psychologists.
0116 254 9568 | www.bps.org.uk

Self Help
Free self-help resources and practical tools based on therapy techniques for managing mental health and wellbeing.
www.getselfhelp.co.uk

Samaritans
Confidential emotional support for anyone in distress or struggling to cope, available 24/7.
116 123 | www.samaritans.org

Age UK
Advice, information, and support services for older people and their families.
0800 678 1602 | www.ageuk.org.uk

Alcoholics Anonymous
Free support groups and information for people who want help to stop drinking.
0800 9177 650 | www.alcoholics-anonymous.org.uk

British Red Cross
Practical support and care services to help people live safely and independently at home.
0344 8711111 | www.redcross.org.uk

Support & Help Directory

NHS Smoking Cessation

Free NHS support, advice, and tools to help people quit smoking.

www.nhs.uk/smokefree

Frank (drugs information)

Confidential advice and information about drugs for individuals, families, and carers.

0300 123 6600 | www.talktofrank.com

Cancer Research UK

Trusted information and support about cancer, treatment, and coping for patients and families.

0300 123 1022 | www.cancerresearchuk.org

Marie Curie Cancer Trust

Care and support for people living with a terminal illness and their families, including end-of-life care and practical advice.

0800 090 2309 | www.mariecurie.org.uk

Macmillan Cancer Support

Information, practical help, and emotional and financial support for people affected by cancer.

0808 808 00 00 | www.macmillan.org.uk

Shelter helpline

Advice and support for people facing housing problems or homelessness.

0808 800 4444 | www.shelter.org.uk

Support & Help Directory

Disabled Living Foundation helpline
Advice on equipment, adaptations, and daily living aids to support independent living.
0300 999 0004 | www.dlf.org.uk

Find a Helpline
A directory to help you find the right helpline for mental health, emotional support, or crisis services.
0300 330 7777 | www.helplines.org

The Lee Spark NF Foundation
A charity for people affected by severe streptococcal infections or necrotising fasciitis.
www.nfsuk.org.uk

The UK Sepsis Trust
A national charity providing information, emotional support, and practical guidance for people affected by sepsis and post-sepsis recovery.
0800 3896255 | www.sepsistrust.org

Citizens Advice
The Citizens Advice service helps people deal with their legal, money and other problems by providing free, independent and confidential advice.
Helpline: 03444 111 444
Website: www.citizensadvice.org

Relaxation Exercises

During and following your time caring for someone in ICU, there are several relaxation exercises you can try out if you feel stressed or overwhelmed. Many have reported that doing these relaxation exercises really help them feel more calm and less anxious while caring for someone in ICU.

Breathing exercise

When we feel anxious, our breathing rate generally increases, making us sweat more, feel dizzy, or get blurred vision. By controlling our breathing, we may decrease feelings of anxiety and make the body feel safe. To control our breathing:

- Sit or lie down in a safe place
- Breathe in for 4 counts, hold for 2 counts, then breathe out for 6 counts
- Continue the above process for 5 to 10 minutes
- You can adjust the counts according to what's most comfortable for you (e.g. 3 counts, 1 count, 5 count)

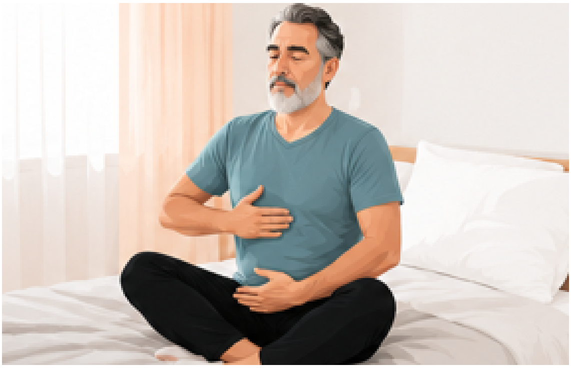

Relaxation Exercises

Imagining your safe place

This exercise is particularly helpful when you are stressed. Imagining that you are in a safe area can help relax your body, decreasing physical symptoms like fast breathing or sweating. To do so:

- Sit down in a safe place
- Close your eyes
- Start relaxing the muscles in your toes, then move up to relaxing your ankles, shin, thighs, and so on until you reach your head
- Once you feel your muscles relaxing, imagine walking or sitting in an area that makes you feel calm. This could be your favourite holiday destination, or at a beach listening to the waves, or walking through the woods.
- Think to yourself: What do you see? What do you hear? What do you smell?
- Take deep breaths and imagine the breeze hitting your face. Stay in this safe place for a few minutes. Enjoy the sensation.
- Slowly open your eyes, keeping this calm feeling and enjoy the peace and quiet.

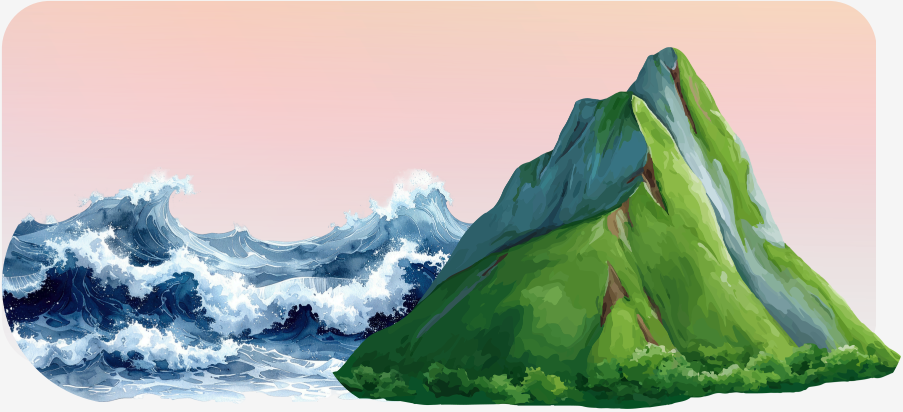

Activity Planning

Activity chart

	Morning	Afternoon	Evening
Monday			
Tuesday			
Wednesday			
Thursday			
Friday			
Saturday			
Sunday			

DNAR

A Do Not Attempt Resuscitation (DNAR) decision is a medical instruction that, if a person's heart or breathing stops, healthcare professionals will not attempt cardiopulmonary resuscitation (CPR).

This decision is made by the clinical team based on the person's overall health, medical condition, and the likelihood of CPR being successful and in their best interests. It does not mean that the person will stop receiving care or treatment – they will still be given all appropriate medical, nursing, and comfort care to support their quality of life.

DNAR decisions are made sensitively, and wherever possible, in discussion with the patient and/or their family or carers. The aim is to ensure that treatment is respectful, compassionate, and avoids interventions that are unlikely to help and may cause more harm than benefit.

If you have any questions or concerns about a DNAR decision, you should speak with the healthcare team, who can explain the reasoning and answer your questions.

Advance Decision

When someone becomes critically unwell, they may be unable to express their wishes, make informed decisions, or communicate decisions about medical treatment at a time it needs to be made. An advance decision (sometimes known as an advance decision to refuse treatment, an ADRT, or a living will) is a way of informing doctors what you do or don't want to happen, before that situation occurs. It lets you choose and explain which medical treatments you do not want to be given by doctors.

If your loved one is thinking about making an advance decision, it is recommended they get advice from the doctors involved in their care. This will help them have all the information they need to make an informed decision. They may also want to get legal advice to help ensure that the decision is clearly and accurately documented.

Once they have come to a decision, your loved one must clearly specify the treatments they are refusing, the reasons, and under what circumstances it applies. Your loved one can change their advanced decision at any time by informing the healthcare team.

Although advisable for an advance decision to be in writing, if the treatment your loved one wants to refuse is not life-sustaining, you can create a valid advance decision simply by telling your doctor which treatments, and under what circumstances you would not want them in the future. An advance decision only needs to be in writing if you want to refuse 'life sustaining' treatment.

Advance Statement

An advance statement (different to advance decision) is a document that your loved one writes themself, or with help from their Lead Professional or family member/friend. This statement is a chance for your loved one to set out any wishes they may have regarding what arrangements they would like to happen if they become extremely unwell. The statement might include things like:

- Their wishes regarding preferred medication
- What treatment has worked well for them in the past and what has not been so helpful
- Any special needs that they may have with regard to diet, health, religion, disability, etc.
- Arrangements that they wish to be made for children/dependants or family pets

If you would like support facilitating conversations regarding any of the above, the following resource might be of help:
The Conversation Project

Organ Donation

Once brain stem death has been confirmed, a doctor may speak with you about next steps. One of the options that may be discussed is organ donation. In the UK, organ donation can proceed after brain stem death has been confirmed. If you know your loved one's wishes regarding organ donation, it's important to share this with the medical team. If you are unsure of their preferences, it's helpful to know that under UK law, organ donation is considered the default unless the individual opted out during their lifetime.

Organ donation nurses

Organ donation nurses provide support to families when organ donation is being considered. They will carefully discuss your loved one's wishes with respect to your own feelings about donation. If donation is agreed, these specialist nurses will coordinate every step, ensuring your family is supported and informed throughout the process. Afterwards, they will continue to offer emotional care, answer any questions you might have, and help you access bereavement support. Please feel free to ask the ICU nurse caring for your loved one if you wish to speak with an organ donation nurse.

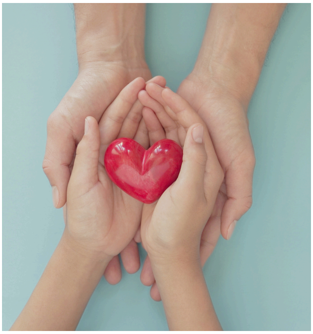

For more details and support:
NHS Organ Donation:
https://www.organdonation.nhs.uk/

Brain Stem Testing

Brain stem testing is one of two ways doctors can certify someone as having died. The other is cardiorespiratory death, which is when breathing and circulation has stopped.

The brain stem is at the very bottom of the brain and controls many functions vital to life such as consciousness, awareness, breathing and the ability to regulate heart rate and blood pressure.

When a person has severe damage to the brain stem (or brain death), the brain cannot send messages to the body to control our unconscious functions, and equally cannot receive messages back from the body. If this is the case, the person will be unable to move or breathe without artificial support through machines. The damage is unfortunately irreversible and the person has no chance of recovery.

What tests are used to confirm brain stem death?
The tests used to determine brain stem death are carried out by two senior doctors at the patient's bedside. They will perform a series of tests twice. All the following criteria need to be met:

- The patient's pupils do not respond to a direct light being shone in their eyes
- The patient's natural eye movements are absent
- The patient's eyes do not blink when the surface of the eyeball is stroked
- The patient does not respond to pain when pressure is applied
- The patient does not breathe when taken off the ventilator
