# Supplementary material for: The Development, Acceptability and Suitability of an Information and Well‐Being Booklet for Family Members of Intensive Care Unit Patients
Source: Nurs Crit Care. 2026 Jul 20;31(4):e70589. doi: 10.1111/nicc.70589 (PMC13385495; doi:10.1111/nicc.70589)
Supplement: Supplementary file 2 — Supporting Information: S1 Table of themes, subthemes and quotes of family members feedback on the booklet. [file NICC-31-0-s002.docx]

**Supplementary material**

**S1.** *Table of themes, subthemes and quotes of family members feedback on the booklet.*

| **Theme** | **Sub-theme** | **Supporting quotes** |
| --- | --- | --- |
| **Information provides reassurance and reduces uncertainty.** | Lack of information | ‘Not knowing what to expect’ (1)  ‘it is overwhelming…when you're going there and you're like, why is that beeping? Why is that making noise? Why is that one doing this?’ (1)  ‘I wanna know what everything is’ (2)  ‘I wish I'd been told all this because I didn't have none of this. Do you know what I mean? And I think that's where all the. I just thought it was me, myself and I getting on with it’ (2)  ‘I just want the truth because so much vagueness and so much uncertainty that you are facing. Every day that those miniature facts begin to feel like you can hold on to something’ (3)  ‘You feel so out of control’ (3)  ‘If this beeps, it's OK…it's normal for the numbers to keep changing… you don't know and you think everything is just like life threatening’ (3)  ‘I had no information whatsoever’ (4)  ‘You're reliant on nurses and wards for all that information…you're rather left out on the edge and you get a bit of information… and then somebody else gives you another bit or contradicts what's been said…you start thinking well, what is the truth?’ (4)  ‘There's so much added information here which had we known we would have gone along a different route’ (4)  ‘I had to Google all of this stuff…had to google about it myself’ (5) |
|  | The booklet as a guiding framework | ‘It gives them more of a how to go forward with things’ (1)  ‘If they have more questions then they can speak to them about that as well, because it's a bit different reasons why people are there, ain't it?’ (1)  ‘I think this would be enough information and then you could go and ask more if you needed to after this’ (2)  ‘What the doctors say sort of goes right over your head. So I think like this is you know this is good. Yeah, I like this because you sort of look for something to guide you while you're going through that’ (5)  ‘Saying what needs to be said and informing people what's going to be happening. And yeah, basically yeah, it gives you a guide of what's gonna be happening’ (5) |
|  | Clear, accessible language | ‘I'm dyslexic…some people might struggle to red and stuff…I understand that do you get what I mean’ (1)  ‘They (doctors) come and tell you this stuff and you're like, what was that? And what does that mean?’ (1)  ‘There's no massive big words, which is really good’ (2)  ‘As simple as possible’ (3)  ‘It's using simple language to describe a difficult task’ (4)  ‘Its very readable’ (4)  ‘It doesn't feel like it's professional’ (5) |
|  | Information about delirium | ‘I'm glad you've put this in because I wasn't told about any of this’ (2)  ‘I think what's important to mention right at the beginning is that it's common for patients in ICU to begin to experience delirium. So you're instantly reassured because otherwise you think your loved one is going to a psychiatric hospital soon because it's like he's the only one experiencing this’ (3)  ‘I didn't know. No one told me’ (3)  'They only explain it when it happens or if it's saving time, but maybe that's why this leaflets might be helpful’ (3)  ‘The more you tell them, the more comfortable they feel, the more normal they feel’ (4)  ‘You wouldn't really have noticed…we didn’t realise at the time… they might be experiencing might be quite severe delirium, but it might not be obvious to you’ (5) |
| **Communication as containment and validation.** | Difficulty asking questions | ‘You know what half the problem is? People don't ask. They think about it afterwards when it's too late, they don't ask’ (2)  ‘I didn't really want to bother people. I just wanted people to do their job’ (2)  ‘If you have any questions, ask. It's not easy to catch the consultants’ (3)  ‘You feel like you're disturbing them (doctors); (3)  ‘You might have a question but fail to ask it because you're terrified you might stop them from doing their job so’ (4)  ‘I just keep my mouth shut because you think one word from me can put somebody's mind off of what they're doing’ (4) |
|  | Lack of communication with consultants | ‘Sometimes when I was talking to them (consultants), they were saying things and I'm like ain't got a clue what you're telling me’ (1)  ‘Hard to even get to talk to one of them’ (1)  ‘I had to sit there for at least five hours just to wait to see the right person, but I didn't know I could ask other people if that makes sense’ (1)  ‘I wanted to talk to the consultant, but sometimes they would be so rushed and it would just be like 2 minutes of an update and I would be disappointed’ (3)  ‘I appreciate that maybe they can't do it every time, but initially I was disappointed’ (3)  ‘There is a new Doctor on a weekly basis’… You might not never see them again and you're building this rapport and then the next week a new one comes in and you start all’ (3) |
|  | Nurses as consistent and trusted points of contact | ‘All the nurses were really good…they did help’ (1)  ‘The intensive care nurses are very knowledgeable…without them, I don't think I would have got by in there…they're the ones that explain’ (2)  ‘The nurse knows everything’ (2)  ‘I don't think the doctors tell you enough, but they're too busy. That's why I suppose the intensive care nurses are there to relay’ (2)  ‘Whenever I asked the nurses in ICU they would explain, they were so lovely’ (5) |
| **Preparation for transitions and recovery beyond ICU** | Lack of preparation about transition to wards. | ‘We didn't know any of this. We just felt a massive change’ (2)  ‘Once you're in a ward, you kind of get forgotten about’ (2)  ‘I'm glad you put that in there because I had to ring up and ask for help because I didn't really know what to do’ (2)  ‘That's a big adjustment because, yeah, because initially Dad thought he was bothered by someone too much in ICU and then he was just left on his own for too long… hearing it initially would be helpful and less stressful’ (3)  ‘She wasn't given any warning that her mental state or that her her lungs were very vulnerable to nearly anything. That that's the only thing I can think of that it would be helpful because my daughter didn't realise and just sort of went on as usual’ (4)  ‘But that transition from ICU to the respiratory ward is horrendous’ (5) |
|  | Lack of preparation about transition home. | ‘You'd be quite shocked at what they give me for you'd be like, is that it? And I'm like, that's it. It was like: You haven't given me any information, really. All you said is it contact us or what can I? You didn't give me anything I could do at home’ (2)  ‘We had no help afterwards at all. And my daughter is now under a counsellor and is in all sorts of problems. And it's purely through lack of knowledge’ (4)  ‘The after bit is really lovely because that's something we knew nothing about’ (4) |
|  | Setting realistic expectations. | ‘It needs to be explained to them that it's not going to be as easy as you think’… thought he was going to come home and thought he was going to carry on as normal and it didn't go like that’ (2)  ‘Every day is like a little milestone’ (1)  ‘celebrating the small milestones’  ‘Why aren't I getting better…all this interior anxiety’ (4) |
| **The Overlooked Burden: Acknowledging Carers’ Wellbeing** | Feeling unseen: carers as the forgotten patient's. | ‘I didn't think there was such a thing as PTSD for people, for the people that watched someone in ICU. I think I was on the verge of that’ (2)  ‘You're really emotionally drained and that matters too’ (3)  ‘It would be really nice to pay more attention, give more attention to the people around the patient’ (3)  ‘I would like this leaflet to kind of have a feel of, carers are important and we see you and we want to hear you from you, because you're not just the carer…it's all about the patient and you feel like you're there for the patient and you end up being a patient yourself but it gets missed. You end up struggling on an emotional level and on a physical level. But just because you're not on the bed you can’t be just dismissed’ (3)  ‘This is difficult for the person who sees and experiences next to bed. It's not only difficult for the person that is experiencing it’ (3) |
|  | Normalising and validating psychological impact. | ‘It's just one of them things, ain't it…its normal’ (to get anxiety) (1)  ‘I've really aged in that couple of months. I've really. Yeah, it took a lot out of me, yeah’ (2)  ‘I didn't go toilet for seven days…because I was so worried about X…I just forgot about what I needed to do and just kept worrying about X’ (2)  ‘It's OK to feel resentful (2)  ‘It's okay not to be okay’(2)  ‘It's like trauma. You feel like you know you're done and then you have a flashback’ (3)  ‘I was splitting into many different parts. I was his advocate slash doctor slash interpreter slash his child slash… it was extremely difficult to remember who I actually am’ (3)  ‘It was the mental bit that got me…you can really go downhill mentally when you have to be put into this sort of situation’ (4) |
|  | Permission to prioritise self-care | ‘I always felt guilty if I weren't there all the time’ (1)  ‘You don't have to be beside their bedside 24 hours’ (2)  ‘You feel like you need to be there 24/7’ (2)  ‘I was glad that I went back to work, actually, because even though it was hard, I remembered who I was, that I had life outside of it, that life still goes on’ (3)  ‘It's OK to go out… You have to live. You have to touch certain points in life that used to mean something to you. You can pause your life for a couple of weeks, but if you pause your life for longer than this, it's really hard’ (3)  ‘It's seeing all of it. It's being there every day. All of it gets into you, into the depths of you, really’ (3)  ‘You don't think about yourself in situations like this’ (4)  ‘When you're a carer…it's just a case of, you know, be kind to yourself. Don't be too hard on yourself… You're only human, you know. You can only do what's possible (4) |
